# Comparison of topical tofacitinib and 0.1% hypochlorous acid in a murine atopic dermatitis model

**DOI:** 10.1186/s40360-018-0232-3

**Published:** 2018-07-03

**Authors:** Tomoki Fukuyama, Sarah Ehling, Jenny Wilzopolski, Wolfgang Bäumer

**Affiliations:** 1 Department of Molecular Biomedical Sciences, College of Veterinary Medicine, North Carolina State University, Raleigh, NC USA; 2 Institute of Pharmacology and Toxicology, Faculty of Veterinary Medicine, Freie Universität Berlin, Koserstraße 20, 14195 Berlin, Germany

**Keywords:** Atopic dermatitis, Dorsal root ganglia, Hypochlorous acid, Tofacitinib, IgE, IL-4, IL-13, NC/Nga mice, Sensory neurons

## Abstract

**Background:**

Topical administration of PR022, 0.05% hypochlorous acid (HOCl) in gel has been demonstrated to be beneficial in a chronic murine atopic dermatitis model. In a follow up study we tested a higher concentration (0.1%) of PR022 HOCl gel in comparison to the Janus kinase inhibitor tofacitinib, both of which are currently in clinical phase studies for treatment of human atopic dermatitis.

**Methods:**

The effect of topically administered HOCl (0.1%) in gel was compared to a topical formulation of tofacitinib (0.5%) in a therapeutic setting on atopic dermatitis-like lesions in NC/Nga mice as well as itch behaviour. NC/Nga mice were sensitized with house dust mite allergen. After reaching visible lesions, mice were treated either topically with HOCl or tofacitinib or gel vehicle for 17 days. After termination of the study, dorsal root ganglia were isolated for ex vivo stimulation and skin samples were taken for cytokine determination in inflamed skin.

**Results:**

When administered onto lesional skin of NC/Nga mice, both HOCl and tofacitinib reduced lesions and scratching behaviour. The reduced inflammatory response by HOCl and tofacitinib treatment was demonstrated by diminished inflammatory cytokines in affected skin tissue from NC/Nga mice. Dorsal root ganglia neurons re-stimulated with a range of mediators of itch showed a reduced response compared to the vehicle control mice, when isolated from tofacitinib or HOCl treated mice.

**Conclusions:**

These data indicate a similar beneficial potential of topical high dose PR022 HOCl (0.1%) in gel and tofacitinib, in a translational murine model of atopic dermatitis.

## Background

Although the incidence of atopic dermatitis (AD) is still increasing, topical pharmaceutical treatment options for AD are limited. Mostly traditional pharmaceutical treatments like topical calcineurin inhibitors or glucocorticoids are still used, but both have dose limiting side effects [1]. Currently there are some small clinical investigations on the use of hypochlorous acid (HOCl) for the topical treatment of AD [2]. It has been reported that HOCl has anti-itch and anti-inflammatory potential in human AD patients [2]. Thus, our group recently performed a detailed preventive and therapeutic intervention study of PR022, HOCl (0.05%) in gel in a chronic mouse model of AD, namely the NC/Nga mice sensitized to a human-relevant allergen (house dust mite allergen). We found a significant inhibition of lesion development and scratching behaviour in the preventive setting and a reduction of lesions and itch in a therapeutic setting accompanied by reduced immunoglobulin E (IgE) response, cytokine secretion in skin and responsiveness of sensory neurons. In vitro experiments with murine bone marrow derived dendritic cells and dorsal root ganglia neurons also revealed an inhibition of MAP kinase activity, inhibition of NFκB pathway and sensory response by pre-incubation of low concentrations of HOCl [3].

Therefore, the present study was performed to test a higher concentration (0.1%) of hypochlorous acid in the NC/Nga mice model in comparison to the Janus kinase (JAK) inhibitor tofacitinib, both of which are currently in clinical studies for treatment of human AD [4]. Apart from efficacy, a further target was to evaluate local tolerability of a higher HOCl concentration.

## Methods

### Materials and reagents

Hypochlorous acid (HOCl) was used as a gel formulation (0.1% AFC, pH 5.5 ± 0.5) and was supplied as “PR022” by Realm Therapeutics, Inc. (Malvern, PA, USA). PR022 is a proprietary formulation of stabilized hypochlorous acid in a gelling agent and an emollient in development for the treatment of atopic dermatitis. In the Phase 2 clinical study, 2 concentrations of PR022 are being evaluated, 0.05 and 0.1% (NCT03351777). For active sensitization, house dust mite allergen (*Dermatophagoides farina,* GREER, Lenoir, NC, USA) was used. Poly-L-lysine, laminin, capsaicin, allyl isothiocyanate (AITC), 2-mercaptoethanol and mineral oil were obtained from Sigma (St. Louis, MO, USA). Dispase was purchased from STEMCELL Technologies Inc. (Cambridge, MA, USA). Fura-2-acetoxymethyl ester (Fura-2 AM), phosphate buffered saline (PBS), collagenase, chloroquine, histamine, and SLIGRL-NH_2_ were ordered from Thermo Fisher Scientific Inc. (Waltham, MA, USA). Fetal bovine serum (FBS), Dulbecco’s modified eagle medium with L-glutamine (DMEM) and RPMI-1640 medium, Ca^2+^ and Mg^2+^-free hank’s balanced salt solution (HBSS), penicillin-streptomycin and fetal bovine serum (FBS) were from Mediatech Inc. (Manassas, VA, USA). MEM eagle (EMEM) medium was obtained from Lonza Group Ltd. (Allendale, NJ, USA). For determination of protein content DCs protein assay kit was used (BIO-RAD, Richmond, CA, USA). The murine IgE ELISA (OptEIA) set was ordered from Becton, Dickinson and Company (Franklin Lakes, NJ). Recombinant mouse interleukin (IL-)1β, IL-31, and tumor necrosis factor (TNF)α were purchased from Pepro Tech, Inc. (Rocky Hill, NJ, USA). Enzyme linked immunosorbent assay (ELISA) kits for IL-1β, IL-4, IL-13, thymic stromal lymphopoietin (TSLP), thymus and activation regulated chemokine (TARC), and TNFα were obtained from R&D systems (Minneapolis, MN, USA). Serotonin and tofacitinib were ordered from Tocris Bioscience (Bristol, UK).

### Mice

NC/Nga (female) mice were ordered from Charles River Japan Laboratories (Tokyo, Japan). The mice arrived at 35 to 40 days of age and were kept in quarantine for at least 1 week. The facility offered a controlled environment (including individually ventilated cages and sentinel animals). The animals were housed at 22 °C with 50% humidity with a 12-h light cycle. The mice were fed with certified pellet diet and received water ad libitum. The study protocol was approved by the Animal Care and Use Committee of the North Carolina State University (IACUC Protocol No. 13–111-B).

### Murine model of atopic dermatitis in NC/Nga mice

Prior to first sensitization, NC/Nga mice were clipped on their back. The following day, mice were sensitized with 30 μl of house dust mite allergen (HDM) suspended (10 mg/ml) in mineral oil and applied topically onto the back twice weekly. To accelerate sensitization, mild tape stripping (“Staples” office tape) was performed weekly just before the first HDM sensitization. Tape stripping was terminated as soon as visible lesions had developed. Treatment of HOCl (0.1% in gel, *n* = 8), tofacitinib (0.5% in lipoderm, *n* = 8) or vehicle (gel, *n* = 8) was started on day 15, where the mice showed a mean lesional score of 2.1. The mice were equally distributed according to their clinical score, that each treatment group represents the mean of 2.1. One group of mice (*n* = 6) was left untreated and served as a base control. The dose selection for tofacitinib was according to a former study in which 0.5% tofacitinib inhibited itch behaviour and inflammation in a mouse model of allergic contact dermatitis, higher doses (when given in acetone) led to irritation of mouse skin, thus it was decided to select a concentration of 0.5% [5].

During the experimental period, back skin thickness, body weight, clinical scores and scratching behavior were monitored once every week. The clinical score was determined as according to the following system: No symptoms, 0; mild, 1; moderate, 2; severe, 3 and extreme, 4. The mean was calculated from the score for erosions, edema, and erythema as well as skin dryness, as described previously [3]. To monitor scratching behaviour mice were video monitored for 30 min directly after sensitization with HDM once a week. Video monitoring was performed with mouse pairs (belonging to the same treatment group) at the same cage. Only repeated strokes with the hindlimb directed to the area of HDM sensitization were counted as scratching bout. Mice were sacrificed by CO_2_ inhalation according to AVMA Guidelines for the Euthanasia of Animals and tissue was collected from each group on day 32.

The back skin, blood and dorsal root ganglia (DRG) were obtained from single mice 1 h after last HOCl or tofacitinib application (this means 24 h after last HDM challenge). Samples were processed (or stored) for IgE determination, histology, cytokine determination and functional measurement of intracellular Ca^2+^ in DRG neurons.

### Histology

Tissue samples from rostral neck skin were excised, fixed in paraformaldehyde (4% solution), sectioned and stained with haematoxylin-eosin. Edema and cell influx were evaluated semiquantitatively (0, no influx, no edema; 1, mild; 2, moderate; and 3, severe influx, severe edema) in a blinded manner in skin sections of each mouse back skin (*n* = 8 for treatment groups, *n* = 6 for untreated control).

### Cytokine determination of skin tissues

One part of the rostral neck skin tissue was snap-frozen in liquid nitrogen. Cytokine determination for skin tissues was performed as described previously [6]. IL-1β, − 4, − 13, TARC, TSLP and TNFα were measured by ELISA. Intra-assay variance was < 10%, inter-assay variance was not calculated as each ELISA (30 samples) was done in one setting (= one plate) for the present study.

### Determination of total IgE in serum

Blood samples collected from single mice was centrifuged at 3000 g to isolate serum. We used an ELSA according to the manufacturer’s protocol to determine total IgE level present in serum.

### Isolation of DRG and calcium imaging on DRG cell culture

Preparation and cultivation of the DRG neurons from the NC/Nga mice was done as described previously [7]. Briefly, DRGs were dissected along the thoracic vertebral column. Single ganglia were digested by means of dispase and collagenase which was dissolved in HBSS for 30 min at 37 °C. A trituration of DRG using fire-polished Pasteur pipettes helped with dissociation. Single cells were centrifuged/washed in DMEM medium containing 10% FBS, resuspended in 50 μl medium and placed onto poly-D-lysine and laminin-coated coverslips. Cells were incubated at 37 °C for 2 h and then flooded with 1 ml of medium and further incubated at 37 °C. Calcium experiments were performed within 24 h of culture.

Changes in intracellular [Ca^2+^] free concentration in single neurons were measured by digital microscopy connected to equipment for ratiometric recording of single cells as described elsewhere [8]. In brief, coverslips attached dorsal root ganglia cells were loaded with fura-2 (8 μmol/L) in DMEM media and incubated at 37 °C for 30 min. For ratiometric imaging, the cells were transferred to a tempered (37 °C) incubation chamber on the stage of the microscope and constantly perfused with Locke solution. Cells that incorporated fura2 were identified using fluorescence microscopy before starting the ratiometric experiment. Cells were marked with region of interest circles and monitored by sequential dual excitation, 340 and 380 nm. The frequency of image acquisition was 100 ms. DRG neurons were initially exposed to IL-1β (1 μg/ml), TNFα (1 μg/ml), histamine (1 mmol/l), followed by IL-31 (1 μg/ml), chloroquine (10 μmol/l), serotonin (1 mmol/l), AITC (100 μmol/l), capsaicin (1 μmol/l) and KCl (75 mmol/l). However, none of the cells were stimulated with more than 3–4 stimuli in the row and the order was switched randomly from slide to slide always ending with KCl. The cells were superfused with a steady Locke solution flux for at least 180 s. after each stimulus as a recovery for the cells prior to the next stimulus.

### Statistical analysis

All data are displayed as mean (±SD). Statistical significance of the difference was estimated at the 5 and 1% levels of probability. If only the significance of differences between mean values of 2 groups were tested, Student t test was used. For comparisons of more than 2 groups we used a one-way ANOVA followed by Dunnett’s multiple comparison test. Comparisons of proportions (study with dorsal root ganglia) were made by means of the Fisher exact test. The data analysis was performed with Prism 4 (GraphPad Software, San Diego, CA, USA).

## Results

### High dose of hypochlorous acid and tofacitinib reduce lesions and itch in the murine NC/Nga model of AD

**Fig. 1:**
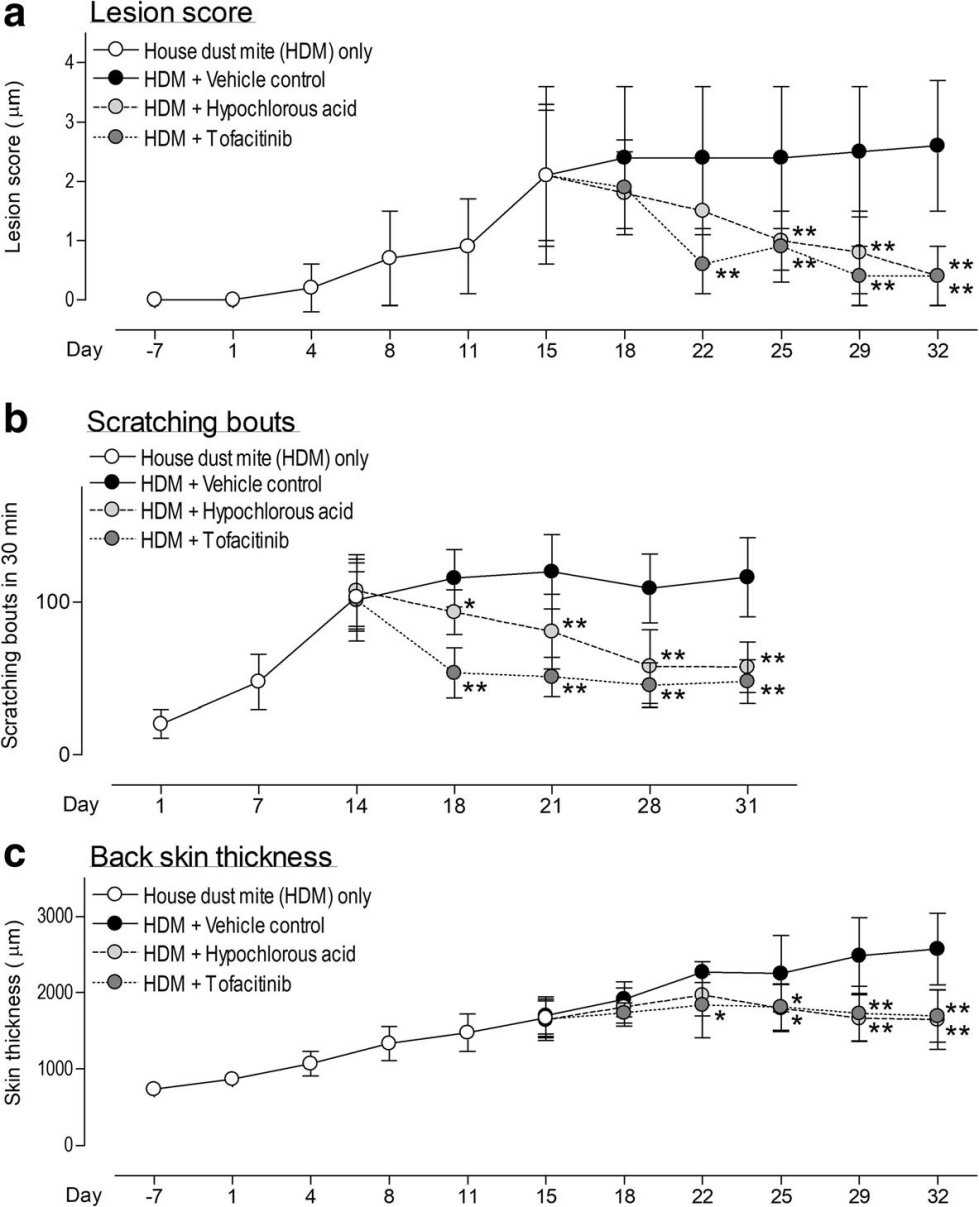
Topical application of tofacitinib and hypochlorous acid, respectively, significantly reduced lesion formation, scratching behaviour and lichenification in NC/Nga mice. **a** Mice developed moderate lesions 15 days after HDM challenge. A therapeutic intervention with hypochlorous acid or tofacitinib significantly reduced the lesion score and hyperplasia at study day 32; **b** Scratching bouts steadily increase during repetitive challenge with house dust mite antigen within the first 14 days. Under therapeutic conditions, hypochlorous acid or tofacitinib significantly reduced scratching behaviour. **c** Back skin thickness was also significantly reduced by treatment with hypochlorous acid or tofacitinib indicating a positive impact on lichenification. *n* = 8 per group, *n* = 6 for untreated control), *p < 0.05, **p < 0.01 compared to vehicle treated mice

To monitor the disease severity of the murine atopic like lesions we scored these according to the four major clinical symptoms of atopic dermatitis, namely excoriations, edema, and erythema and skin dryness. After starting HDM application, the lesional score gradually increased in the vehicle group starting from day 6. Topical administration of 0.1% HOCl and 0.5% tofacitinib resulted in reduction in skin lesions compared to the vehicle-only group. This difference became significant 10 days after initiation of HOCl treatment and 6 days after tofacitinib treatment initiation (Fig. 1a). In analogy to the lesion severity scratching bouts steadily increased throughout the study in the vehicle control group. This scratching behaviour was significantly reduced by topical administration of HOCl or tofacitinib compared to that of vehicle control group (Fig. 1b). A decrease of back skin thickness was observed in both treatment groups indicating a reduced lichenification (Fig. 1c). There was no difference in bodyweight in vehicle control, tofacitinib and hypochlorous acid gel indicating a lack of systemic effect of these two topical treatment options (data not shown).

**Fig. 2:**
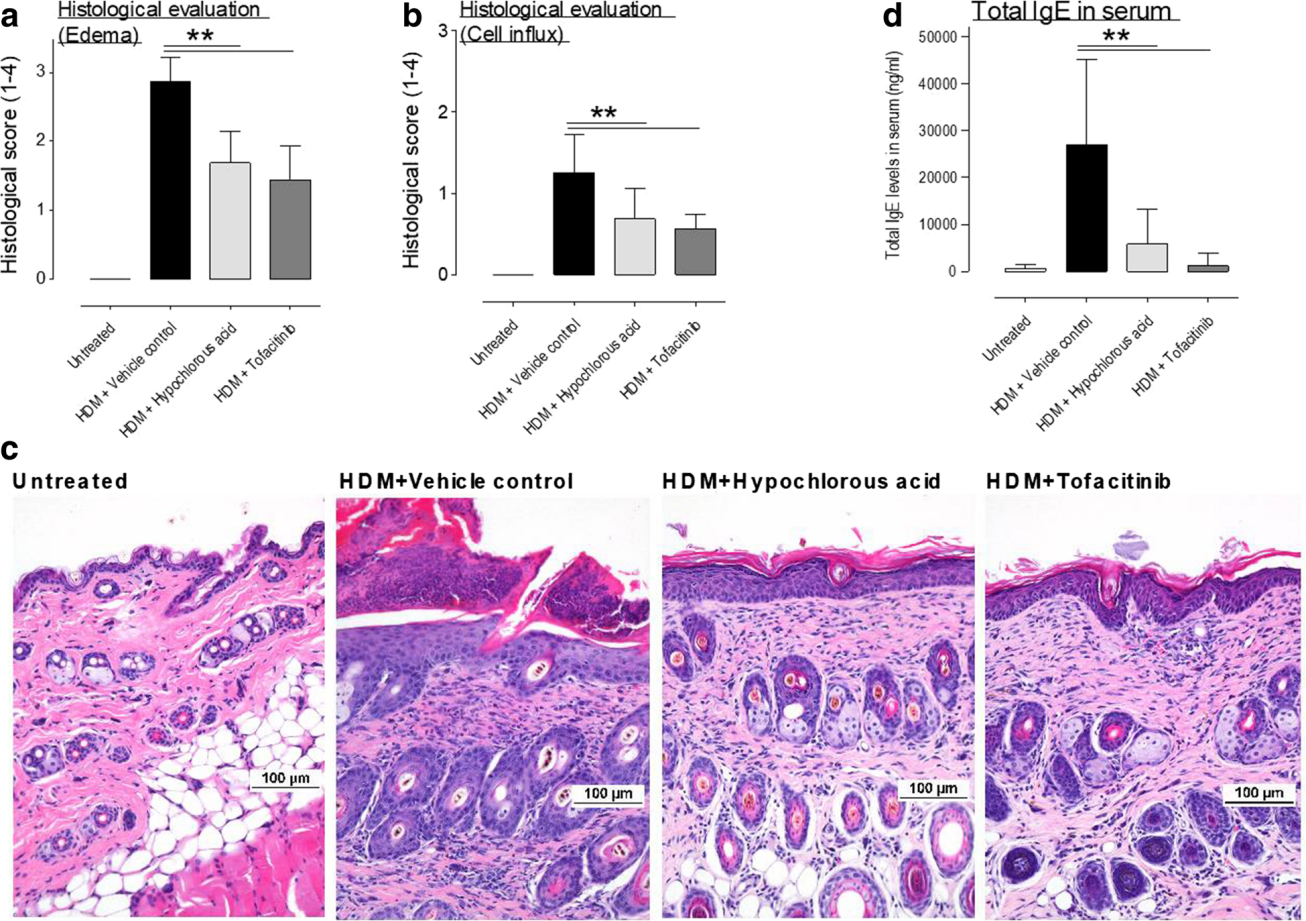
Hypochlorous acid and tofacitinib significantly reduced edema and inflammatory cell influx as well as total IgE in serum. **a** NC/Nga mice were sacrificed on study day 32 and histological samples were taken for the detrermination of edema and inflammatory cell influx. Both treatment options led to significantly reduced edema (**a**, **c**) and inflammatory cell influx (**b**, **c**) compared to vehicle treated and HDM challenged mice. **d** Whereas a HDM challenge led to a vast increase of total IgE in vehicle treated mice, this concentration was significantly reduced by topical treatment with HOCl or tofacitnib., n = 8 per group, *n* = 6 for untreated control), *p < 0.05, **p < 0.01 compared to vehicle treated mice

The histological evaluation confirmed the in vivo findings: At day 32 vehicle treated mice had markedly increased edema formation and inflammatory cell influx. This was significantly reduced by topical treatment with HOCl and tofacitinib compared to vehicle treatment (Fig. 2a and b).

As a more objective measure of allergic inflammation in the skin and to corroborate histological findings, tissue cytokines were determined in back skin. In addition to an impact on pleiotropic pro-inflammatory cytokines like IL-1β, and TNFα, the level of classical Th2 cytokines like TARC, IL-4 and IL-13 was strongly diminished by treatment with hypochlorous acid gel or tofacitinib compared to the vehicle control treatment in back skin. In addition, the cytokine TSLP which has been described to be a direct pruritogen, was also significantly reduced (Table 1). By direct comparison of reduction of cytokines by tofacitinib and HOCl it seems that tofacitinib was more potent in reducing the cytokines (significantly for IL-4).

**Table 1:** Cytokine production in back skin of topical application of hypochlorous acid or tofacitinib in NC/Nga mice

Group	Untreated	HDM + Vehicle control	HDM + Hypochlorous acid	HDM + Tofacitinib
IL-1β (pg/mg)	84 ± 25	140 ± 60	83 ± 27 *	41 ± 17 **
IL-4 (pg/mg)	32 ± 18	157 ± 57	87 ± 28 **	36 ± 19 **†
IL-13 (pg/mg)	183 ± 101	739 ± 451	321 ± 68 *	162 ± 76 **
TARC (pg/mg)	32 ± 16	100 ± 49	53 ± 12 *	27 ± 10 **
TNFα (pg/mg)	98 ± 50	453 ± 230	207 ± 37 **	108 ± 40 **
TSLP (pg/mg)	69 ± 19	186 ± 96	115 ± 26 **	76 ± 25 **

Back skin tissue was collected 24 h after last HDM challenge and 1 h after last HOCl or tofacitinib application. Results are expressed as mean ± S.D. (pg/mg protein; *n* = 8 per group, *n* = 6 for untreated group)

*: *P* < 0.05 and **: *P* < 0.01 (Dunnett’s multiple comparison test) vs. HDM + vehicle control group. †: *P* < 0.05 (Tukey’s Multiple Comparison Test) HDM + Tofacitinib vs. HDM + Hypochlorous acid group

Total IgE was vastly increased compared to untreated age matched mice after chronic treatment with HDM. This increase was significantly reduced by topical administration of hypochlorous acid gel or tofacitinib compared to vehicle control treatment (Fig. 2c).

### The ex-vivo-response of sensory neurons to different pruritus stimuli is diminished after HOCl or tofacitinib treatment

**Fig. 3:**
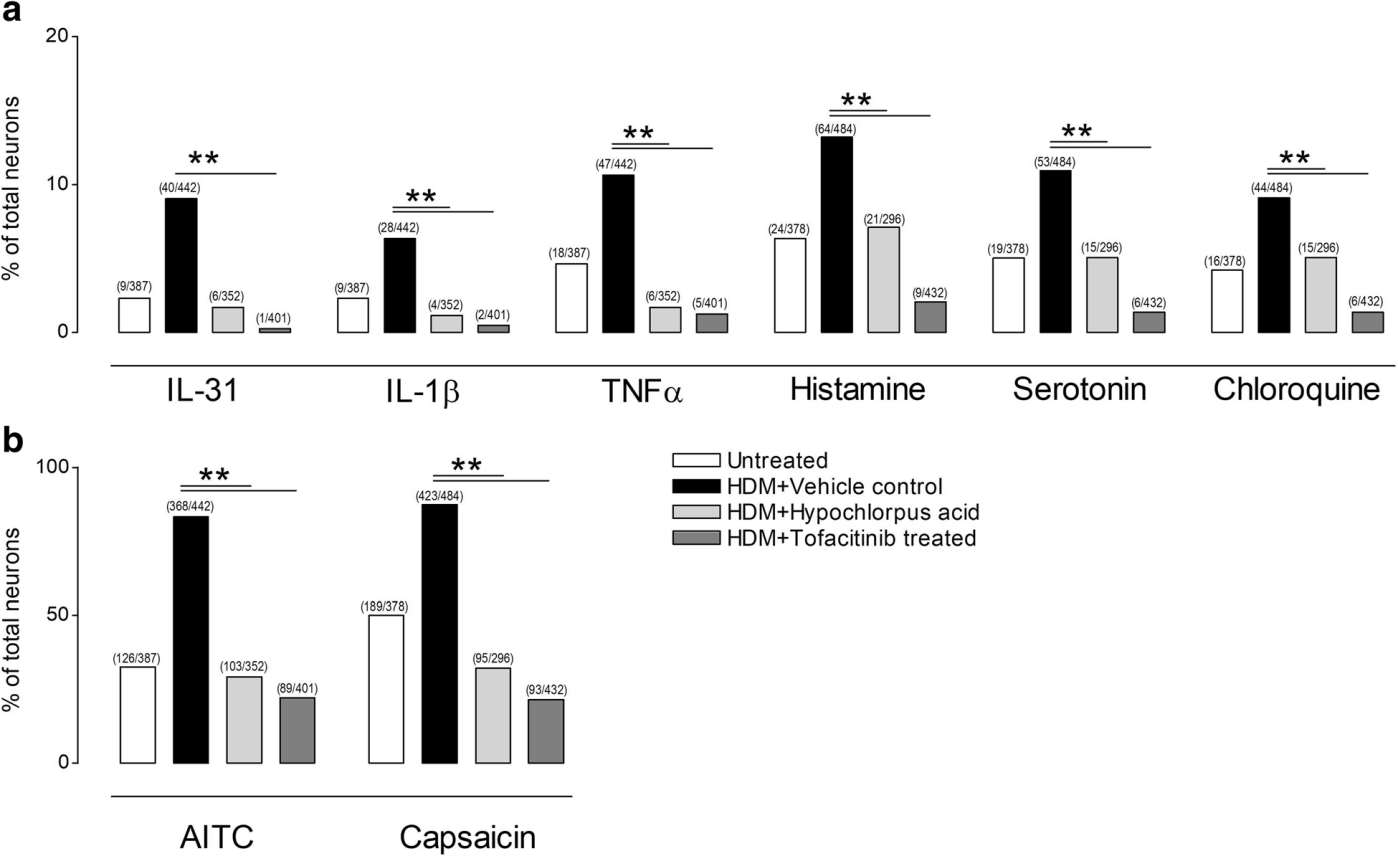
Ex vivo response of sensory neurons stimulated with different pruritogens. **a** The ex vivo response of DRG neurons to IL-31 (1 μg/ml), IL-1β (1 μg/ml), TNFα (1 μg/ml), histamine (1 mmol/l), serotonin (1 mmol/l) and chloroquine (10 μmol/l) and (**b**) AITC (100 μmol/l) as well as capsaicin (1 μmol/l) were compared to DRG obtained from age matched vehicle treated and house dust mite antigen sensitized and challenged NC/Nga mice. The sensory nerve activation in dorsal root ganglia isolated from hypochlorous acid or tofacitinib treated mice were reduced in response to all stimuli compared to vehicle treated. Absolute number of neurons in brackets, **p < 0.01 compared to vehicle treated mice

Both, HOCl and tofacitinib application had a significant effect on itch behavior. Thus, in a next step we assessed whether this response could also be retraced on dorsal root ganglia (DRG) ex vivo. For this, DRG were obtained from NC/Nga mice treated with vehicle, HOCl gel or tofacitinb and the ex vivo response of DRG neurons to histamine and non-histaminergic stimuli (serotonin, chloroquine, IL-31) were compared to DRG obtained from vehicle treated, house dust mite sensitized mice and a basal control (i.e. untreated, age matched NC/Nga mice). When DRG of hypochlorous acid gel or tofacitinib treated mice were monitored, histamine as well as non-histaminergic stimuli led to significant less sensory nerve activation compared to vehicle treated mice (Fig. 3a). In addition, we observed that an enhanced response to AITC (TRPA1 channel activator) as well as capsaicin (TRPV1 channel activator) was also reduced, which can be interpreted as a sign of reduced peripheral sensitization (Fig. 3b).

## Discussion

Atopic dermatitis (AD), a multifactorial allergic-inflammatory disease with complex pathophysiology, remains incurable and affects 10 to 20% of children [9, 10]. The two major symptoms of AD are pruritus (itch) and inflammatory lesions. Topical treatment options are limited and the frequently used glucocorticoids and calcineurin inhibitors have side effects, particularly with their long-term use [11].

In the present study employing the NC/Nga mouse model of AD, topical treatment with 0.1% hypochlorous acid showed a therapeutic effect in an established atopic dermatitis mouse model, significantly reducing both lesions and allergic itch throughout the experimental period. Results showed HOCl gel to have equivalent efficacy to the topical JAK inhibitor tofacitinib in reducing clinical manifestations of the disease (such as lesion score) as well as reducing the itch response. Although the reduction sets in faster in the tofacitinib group, the overall effect on day 31 were comparable. Tofacitinib was chosen as a comparator, as first results of a clinical phase II study revealed a great benefit of this topically administered JAK inhibitor. As a glucocorticoid was chosen as comparator in a former study, this offers the comparison with another topical therapeutic with a different mechanism of action.

In the present study we used a gel that is formulated at a pH range 6 (+/− 0.5). In this specific formulation available free chlorine is present in the form of hypochlorous acid at a concentration of ca. 0.1% (1000 ppm), a concentration which displayed no safety concerns in mice in this study. Although no side-by-side comparison has been performed, compared to the former study, the onset of reduction of itch and lesion score seems faster, when HOCl is formulated as 0.1% compared to 0.05% [3]. It took 17 treatment days to reduce the lesion score to > 50% compared to vehicle in the former study [3], whereas more than 50% reduction was already achieved after 10 treatment days in the current study.

During the study, a steady increase of scratching behavior was observed within the first 14 days of sensitization with house dust mite antigen (Fig. 1b). Comparable to the former study, we again observed a higher responsiveness to pruritic stimuli ex vivo in comparison to non-sensitized and age matched NC/Nga mice. The sensory neurons excised from vehicle treated and house dust mite antigen sensitized and challenged NC/Nga mice were generally more responsive to stimuli like IL-31, IL-1β, TNFα, histamine, chloroquine, serotonin, capsaicin and AITC. These results can be interpreted as a general state of peripheral sensitization often seen with stages of chronic itch [12]. Both therapeutic options (HOCl as well as tofacitinib) let to a significant reduction of this hyper responsiveness. These results can be interpreted as a reduction or normalization of sensory neurotransmission, which finds its in vivo correlation in the significant reduction of scratching behaviour. A limitation of this study is that the number of mice taken for each group do not allow a non-inferiority comparison between HOCl and tofacitinib (which was not the intention of this study).Thus, a comparison of the inhibitory effect can only be made descriptively. Nevertheless, compared to vehicle treated mice, both treatment options showed significant reduction of itch and lesions at the end of the study.

NC/Nga mice are frequently used for mechanistic studies as well as for testing new therapeutic options of AD [3, 13]. The advantage of this model is that lesions are induced with a relevant allergen (house dust mite antigen) and the phenotype is quite translational to the human counterpart (constant itch, Th2 cytokines, lichenification and enhanced IgE levels) [14]. Taken together, it seems that even a repeated/chronic administration of a hydrogel containing 0.1% available free chlorine hypochlorous acid is well tolerated and reveals significant anti-inflammatory and anti-itch properties, comparable to a JAK inhibitor (tofacitinib) which is currently in clinical phase III for the treatment of human AD. Reduced inflammation was confirmed histologically and by reduced concentrations of cytokines, which are frequently associated with atopic dermatitis (IL-4, IL-13, TARC [9]) and cytokines for which a major role in the mediation of itch has been described (TSLP [15]).

## Conclusions

A repeated/chronic administration of a hydrogel containing 0.1% available free chlorine hypochlorous acid is well tolerated and reveals significant anti-inflammatory and anti-itch properties, comparable to a JAK inhibitor (tofacitinib) which is currently in clinical phase III for the treatment of human AD.

## Abbreviations

AD: Atopic dermatitis
AITC: Allyl isothiocyanate
DRG: Dorsal root ganglia
ELISA: Enzyme linked immunosorbent assay
FBS: Fetal bovine serum
Fura-2 AM: Fura-2-acetoxymethyl ester
HBSS: Hank’s balanced salt solution
HDM: House dust mite allergen
HOCl: Hypochlorous acid
IgE: Immunoglobulin E
IL: Interleukin
JAK: Janus kinase
KCl: Sodium chloride
ms: Millisecond
NFκB: Nuclear factor κB,
nm: Nanometer
PBS: Phosphate buffered saline
TARC: Thymus and activation regulated chemokine
TNFα: Tumor necrosis factor alpha
TSLP: Thymic stromal lymphopoietin
